# Physical activity restriction in age-related eye disease: a cross-sectional study exploring fear of falling as a potential mediator

**DOI:** 10.1186/s12877-015-0062-8

**Published:** 2015-06-12

**Authors:** Angeline M. Nguyen, Karun S. Arora, Bonnielin K. Swenor, David S. Friedman, Pradeep Y. Ramulu

**Affiliations:** Wilmer Eye Institute, Johns Hopkins University, 600 North Wolfe Street, Maumenee B-110, Baltimore, MD 21287 USA; Johns Hopkins Bloomberg School of Public Health, Baltimore, MD USA; The Dana Center for Preventive Ophthalmology, Wilmer Eye Institute, 600 North Wolfe Street, Maumenee B-110, Baltimore, MD 21287 USA

**Keywords:** Glaucoma, Age-related macular degeneration, Fear of falling, Physical activity

## Abstract

**Background:**

Fear of falling (FoF) is predictive of decreased physical activity. This study sought to determine if FoF mediates the relationship between decreased vision and physical activity restriction in individuals with glaucoma and age-related macular degeneration (AMD).

**Methods:**

Accelerometers were used to measure physical activity over 1 week in 59 control, 83 glaucoma, and 58 AMD subjects. Subjects completed the University of Illinois at Chicago Fear of Falling Questionnaire, and the extent of FoF was estimated using Rasch analysis. In negative binomial models adjusting for demographic, health, and social factors, FoF was investigated as a potential mediator between the severity of visual field (VF) loss (in glaucoma patients) or the severity of contrast sensitivity (CS) loss (in AMD patients) and decreased engagement in physical activity, defined as minutes spent in moderate-to-vigorous physical activity (MVPA) per day.

**Results:**

In multivariate negative binomial regression models, 5-decibels worse VF mean deviation was associated with 26 % less engagement in MVPA [rate ratio (RR) = 0.74, p < 0.01] amongst glaucoma subjects. When FoF was added to the model, the RR increased from 0.74 to 0.78, and VF loss severity remained associated with less MVPA at a statistically significant level (p < 0.01). Likewise, 0.1 log units worse CS was associated with 11 % less daily MVPA (RR = 0.89, p < 0.01) amongst AMD subjects. When FoF was added to the model, the RR increased from 0.89 to 1.02, and CS loss was no longer associated with MVPA at a statistically significant level (p = 0.53).

**Conclusions:**

FoF may mediate the relationship between vision loss and physical activity restriction amongst patients with AMD. Future work should determine optimal strategies for reducing FoF in individuals with vision loss in order to prevent the deleterious effects of physical activity restriction.

**Electronic supplementary material:**

The online version of this article (doi:10.1186/s12877-015-0062-8) contains supplementary material, which is available to authorized users.

## Background

Individuals with vision loss have substantial limitations in physical activity [1–4], and low levels of physical activity are associated with lower quality of life, greater morbidity, and higher mortality rates [5–11]. Fear of falling (FoF), defined as a low perceived ability to avoid falling during routine activities of daily living, [12] is a plausible intermediary in the pathway between age-related vision loss and decreased physical activity. Vision loss is associated with greater fear of falling (FoF) [13–17], and studies have pinpointed visual field (VF) loss and decreased contrast sensitivity (CS) as the most significant visual predictors of FoF in glaucoma and age-related macular degeneration (AMD) patients, respectively [16, 17]. FoF may result from the true increased risk of falls [18, 19], decreased balance [20, 21], and greater likelihood of bumping into objects [22] noted in these patients. Studies using questionnaires to estimate level of physical activity such as the Short Form 36 (SF-36) Health Survey or the Yale Physical Activity Survey have shown that FoF is associated with a decrease in physical activity and physical health [23–25]. To further study the association between vision and decreased physical activity [3, 26, 27], one prior study which sought to determine if FoF is a driver of physical activity restriction in individuals with vision loss found that those with eye disease were more likely to report activity restriction as a result of FoF than normally-sighted individuals [28]. This study, however, did not define whether the activities restricted related to physical activity or to other activities of daily living, and the study conclusions about mediation were based upon self-report of activity restriction alone.

A better understanding of the specific factor(s) along the pathway from vision loss to physical activity limitation will help in developing interventions to avoid the deleterious effects of physical activity restriction. We speculate that FoF resulting from different types of vision may cause or exacerbate physical activity restriction. Using novel, direct measures of FoF (Rasch-analyzed responses to a validated 16-item questionnaire about FoF) [29] and physical activity (waistband omnidirectional accelerometers) [30], we tested the hypothesis that FoF is a partial mediator for the relationship between vision loss and decreased physical activity in individuals with glaucoma and AMD, the two most common causes of irreversible vision loss in the United States [31].

## Methods

The study protocol adhered to the tenets of the Declaration of Helsinki and was approved by the Johns Hopkins Medicine Institutional Review Board. All participants provided written informed consent and completed study procedures between July 2009 and June 2012.

### Study subjects

Subjects were recruited from a convenience sample of patients at the Johns Hopkins Wilmer Eye Institute. Patients were eligible if they were 60 to 80 years old and able to communicate in English. Patients were ineligible if they had a history of an ocular laser procedure in the prior week, non-ocular surgery or hospitalization in the prior 2 weeks, or ocular surgery in the prior 2 months. The rationale and criteria used to define the three study groups have been previously described in detail [3, 17, 32].

Control and glaucoma subjects both completed Humphrey 24-2 VF testing (Carl Zeiss Meditec, Dublin, CA) within 12 months of the study. Control subjects had a diagnosis of glaucoma suspect or ocular hypertension based on optic nerve and VF findings, and had a presenting visual acuity (VA) better than 20/40 in both eyes, a mean deviation (MD) better than -3 dB in at least one eye using the Swedish interactive thresholding algorithm (SITA) standard Humphrey 24-2 VF test, and a glaucoma hemifield test (GHT) result other than “Outside Normal Limits” in both eyes. Glaucoma subjects had a diagnosis of primary open angle, primary angle closure, pseudoexfoliation, or pigment dispersion glaucoma based on optic nerve and VF findings. Additionally, they had a better-eye MD equal to or worse than -3 dB with a GHT result of “Outside Normal Limits”, “Borderline”, or “Generalized Reduction in Sensitivity” in both eyes. AMD subjects had bilateral AMD with evidence of drusen, geographic atrophy, or choroidal neovascularization in both eyes. VA in AMD subjects was required to be 20/32 or worse in both eyes, or worse than 20/200 in one eye.

### Measurement of physical activity

Physical activity was assessed for 7 days of regular activity [33] using a waistband omnidirectional accelerometer (Actical; Respironics, Inc, Adover, MA). Subjects were instructed to clip the tracking device to their waistband near their hip during all waking hours except while in water. The accelerometers were set to record activity in 1-minute epochs over the full study period. Activity was quantified as steps taken over that 1-minute period, and also quantified by the intensity of motion during that one minute interval as “counts”, which reflect a transformation of total acceleration into an arbitrary unit. Count data was used to categorize physical activity occurring over each study minute as sedentary, light, moderate, or vigorous based on the cut-points defined by Colley and Tremblay [34]. Further details regarding the function and validity of the device are described in detail elsewhere [3].

Subjects were queried directly about adherence to device-wearing during phone calls, and days in which devices were not worn were excluded from analyses. Valid device-wear was also assessed by estimating accelerometer wear-time, defined as the interval between the first and last minutes with non-zero counts for each study day, and days of accelerometer data with fewer than 8 hours of estimated wear-time were excluded [35]. Individuals with fewer than 2 valid study days were excluded from all analyses.

### Measurement of potential mediators

FoF was measured using the University of Illinois at Chicago Fear of Falling Measure, a previously validated questionnaire (see Additional file 1) [29]. Questions were administered orally to subjects during an in-person interview, which asked about how worried they would be if they were to perform each of 16 different tasks. One of four possible responses was accepted for each task: not worried, a little worried, moderately worried, or very worried. Moderately or a little worried were combined into a single category, as previously described [29]. The responses to the questions about FoF were then analyzed in a Rasch model using Winsteps (Winsteps, Chicago, IL) to estimate linear item measures for each task and linear person measures for each participant. Both item measure and person measures were expressed in log-odds units, or logits, along the same scale. Subjects were allotted higher person measures if they indicated that they are able to perform more difficult tasks without FoF. Items were allotted higher item measures if the item could be performed without FoF by subjects with greater ability. All control, glaucoma, and AMD subjects were included in the same Rasch model, which allowed for direct comparison of person measures between groups. The subjects having person measures in the lowest tertile were categorized as having moderate-to-severe FoF, while those with person measures in the highest two tertiles were categorized as having absent-to-mild FoF.

We hypothesized that FoF would mediate the association between vision loss and physical activity, which would be manifested by attenuation of the association between vision loss and physical activity in regression analyses. To test the specificity of FoF as a mediator, we also assessed driving status to determine if it could also act as a potential mediator between decreased vision and physical activity, since driving is a primary method for leaving the home and rates of physical activity have been shown to be higher outside the home than inside the home [3]. Driving status was evaluated with a questionnaire adapted from the Salisbury Eye Evaluation Driving Study (SEEDS) [36]. Subjects were asked if they have driven a car in the past 3 months in order to assess driving cessation. Subject who had not ceased driving were considered to have limited driving if they (1) had driven less than a total of 3,000 miles during the past year, or if in the past 3 months they (2) have not driven to unfamiliar areas, or (3) driven in at night.

### Measurement of vision and other covariates

VA was assessed with habitual correction using the Early Treatment of Diabetic Retinopathy Study (EDTRS) chart at either 1 or 4 meters. Better-eye acuity was transformed to logarithm of the minimum angle of resolution (logMAR) units for analysis [37]. Eyes with VAs of count fingers and hand movements were assigned logMAR acuities of 1.8 and 2.3 respectively [38]. CS was measured under binocular conditions as the number of letters read correctly on the Pelli-Robson chart and converted to a log scale (logCS) [39]. Lenticular changes, including nuclear sclerotic, cortical, posterior subcapsular changes, or posterior capsular opacification (PCO) in pseudophakic eyes, were graded after pupillary dilation as being present or absent as described previously [17].

Standardized questionnaires were used to collect demographic information, including age, sex, race, education, employment, and living arrangements. Height and weight were measured directly to calculate body mass index (BMI). Grip strength was assessed using a Jamar hand-held dynamometer (Sammons Preston, Inc, Bolingbrook, IL) with strength recorded as the mean in kilograms of force of 3 consecutive trials using the dominant hand. Comorbid illnesses were evaluated using a standardized questionnaire [40]. Depressive symptoms were evaluated with the short form of the Geriatric Depression Scale [41]. Cognitive ability was evaluated using the Mini Mental State Exam (MMSE) for the visually impaired [42].

### Statistical methods and programming

Group differences in demographic, health, and vision characteristics were analyzed using the Student’s t-test or Kruskal-Wallis test for continuous variables and χ2 for categorical variables using Stata software version 13 (Stata Corp., College Station, TX).

Patient characteristics impacting time spent in moderate-to-vigorous physical activity (MVPA) each day were modeled using negative binomial models, which express the relationship of patient characteristics to the time spent in MVPA as rate ratios (RRs) and 95 % confidence intervals (CI). For all the negative binomial analyses in this study, each person-day was analyzed as a separate observation and generalized estimating equations were used to account for correlation of physical activity measures across different study days of the same subject.

Analyses were first performed to determine which measures of visual functioning were associated with MVPA in the glaucoma and AMD groups. VF loss and CS were predictive of decreased MVPA in glaucoma and AMD subjects, respectively. Therefore, the analyses below were each performed for glaucoma and AMD separately with the VF loss and CS as the marker of disease severity for each group, respectively.

Separate univariate negative binomial analyses were performed with MVPA as the dependent variable and visual and non-visual characteristics as the independent variables in order to identify covariates to be further explored in multivariate analyses (Tables 2 and 3 Model 0). Covariates found to be statistically significant in their association with both the visual variables (CS in AMD or VF loss in glaucoma) and MVPA were included in multivariate analyses. Multivariate negative binomial analyses were performed to examine the relationship of VF loss and CS with MVPA (Tables 2 and 3 Model 1). Covariates included in multivariate models were age, sex, race, education, and number of comorbid diseases, each of which were previously demonstrated to affect physical activity levels in prior studies [43]. Depression, employment, and BMI were not included in these models, as it was not clear whether these factors served as a risk factor for decreased physical activity or as a consequence thereof [44–47].

To demonstrate that VF loss and CS are associated with engagement in MVPA in glaucoma and AMD, respectively, negative binomial models were constructed in which each person-day was analyzed as a separate observation and generalized estimating equations were used to account for correlation of physical activity measures across different study days of the same subject. To assess potential mediation, the relationship between VF loss or CS and MVPA was assessed using negative binomial regression analysis via Baron and Kenny’s step-wise approach [48]. To do so, FoF and driving were each separately added to the multivariate models to determine if the association between VF loss or CS and MVPA was attenuated in these extended models (Tables 2 and 3 Models 2 and 3).

## Results

Fifty-nine control, 83 glaucoma, and 58 AMD subjects were enrolled in the study and included in the analysis on the basis of having at least 2 valid study days. Included control subjects had an average of 6.69 days of valid device-wear, compared to 6.74 days for glaucoma subjects (p = 0.81), and 6.22 days for AMD subjects (p = 0.08). Neither glaucoma nor AMD subjects differed significantly from control subjects with regard to sex, education, employment, living arrangements, BMI, grip strength, number of comorbid medical conditions, depressive symptoms, or cognitive ability (p > 0.05 for all) (Table 1).

**Table 1:** Characteristics of study participants by disease status

	Controls (n = 59)	Glaucoma (n = 83)	AMD (n = 58)
**Vision**			
^a^Better-eye VA logMAR, median (IQR)	0.08 (0.00, 0.16)	**0.16 (0.08, 0.34)**	**0.38 (0.20, 0.64)**
^a^Binocular log CS, median (IQR)	1.85 (1.80, 1.98)	**1.50 (1.35, 1.75)**	**1.50 (1.35, 1.60)**
^a^Better-eye VF MD, median (IQR)	0.17 (0.90, -0.65)	−**7.96 (-4.81, -16.46)**	--
^b^No. with cataract/PCO either eye, N (%)	12 (21.8)	31 (37.4)	18 (33.3)
^b^No. with cataract/PCO both eyes, N (%)	6 (10.7)	12 (14.5)	8 (14.8)
**Demographics**			
^a^Age in years, median (IQR)	69.4 (65.2, 72.8)	70.4 (66.4, 74.5)	**75.8 (71.0, 78.3)**
^b^Ethnicity, N (%)			
White non-Hispanic	45 (75.0)	46 (55.4)	**57 (90.5)**
White-Hispanic	1 (1.7)	6 (7.2)	**4 (6.4)**
African-American	12 (20.0)	27 (32.5)	**1 (1.6)**
Asian	2 (3.3)	4 (4.8)	**1 (1.6)**
^b^Female, N (%)	37 (61.7)	44 (53.0)	36 (57.1)
^a^Education in years, median (IQR)	16.5 (14.0, 17.0)	16.0 (14.0, 17.0)	16 (13.0, 17.0)
^b^Employed, N (%)	23 (38.3)	35 (42.2)	14 (22.2)
^b^Living alone, N (%)	11 (18.3)	16 (19.3)	14 (22.2)
**Health/cognition**			
^a^Body mass index in kg/m^2^, median (IQR)	27.9 (23.8, 32.2)	28.0 (24.5, 32.2)	27.5 (24.6, 32.5)
^a^Grip strength in kg, median (IQR)	26.3 (21.3, 32.3)	28.7 (21.2, 36.7)	27.0 (21.3, 34.3)
^a^No. comorbid illnesses, N (IQR)	2 (1, 3)	2 (1, 3)	2 (1, 4)
^b^No. depressive symptoms, N (%)	3 (5.0)	5 (6.0)	3 (4.8)
^a^MMSE-VI score as #/22, N(IQR)	21 (20, 22)	21 (20, 22)	21 (20, 22)

*AMD* = age-related macular degeneration; *logMAR* = logarithm of the minimum angle of resolution; *IQR* = interquartile range; *VA* = Visual Acuity; *VF MD* = Visual field mean deviation; *PCO* = Posterior Capsular Opacification; *No*. = number; MMSE-*VI* = Mini-mental status examination for the visually impaired

Values in bold indicate p < 0.05

^a^Kruskal-wallis test

^b^Chi-squared test

AMD, but not glaucoma, subjects were older than controls (p < 0.001). AMD subjects were more often white compared to controls (p = 0.06). Better-eye VA was worse in both glaucoma subjects (median logMAR acuity 0.16, interquartile range [IQR] = 0.08 to 0.34) and AMD subjects (median logMAR acuity = 0.38, IQR = 0.20 to 0.64) as compared to controls (median logMAR = 0.08, IQR = 0.00 to 0.16) (p < 0.001 for both). Binocular log CS was worse in both glaucoma subjects (median log CS = 1.50, IQR = 1.35 to 1.75) and AMD subjects (median log CS = 1.50, IQR = 1.35 to 1.60) as compared to controls (median log CS = 1.85, IQR = 1.80 to 1.98) (p < 0.001 for both). Glaucoma subjects had greater VF loss than control subjects with a median better-eye MD of -8.0 dB (IQR = -16.5 to -4.8 dB) versus a median of 0.2 dB (IQR = -0.7 to 0.9) for control subjects (p < 0.001).

**Fig. 1:**
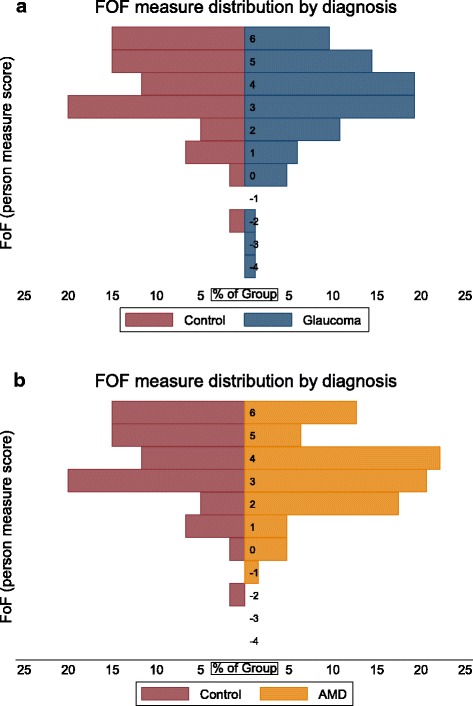
Distribution of FoF in **a** control subjects versus glaucoma subjects and **b** control subjects versus AMD subjects. Fear of falling is presented as Rasch-derived person measures based on participants’ responses to the University of Illinois at Chicago Fear of Falling Questionnaire. A lower fear of falling person measure indicates greater fear of falling in the individual

**Fig. 2:**
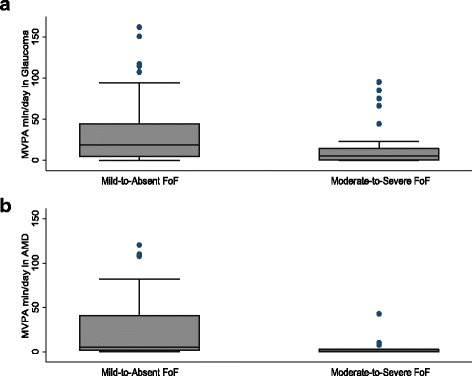
Box-whiskers plots of **a** minutes spent in MVPA per day for glaucoma subjects and **b** minutes spent in MVPA per day for AMD subjects. Box-whiskers plots show the 25^th^ and 75^th^ percentile range (box) and median values (transverse lines in the box)

A greater proportion of both the glaucoma and AMD groups had low FoF person measures (greater fear of falling) as compared to controls (Fig. 1). For glaucoma subjects, median time spent engaged in MVPA was 19.7 minutes/day (IQR = 4.5 to 50.1 minutes/day) for those with mild-to-absent FoF, as compared with 5.0 minutes/day (IQR = 0.4 to 14.6 minutes/day; p < 0.01) for those with moderate-to-severe FoF (Fig. 2a). For AMD subjects, median time spent engaged in MVPA was 6 minutes/day among subjects with mild-to-absent FoF (IQR = 1.7 to 41.5 minutes/day), as compared with 0.5 minutes/day (IQR = 0.0 to 2.8 minutes/day p < 0.01) for those with moderate-to-severe FoF (Fig. 2b).

Greater VF loss was associated with less MVPA amongst glaucoma subjects in univariate and multivariate models (Table 2, model 0 and 1). To determine if FoF or driving status could at least partially explain the relationship between VF loss and reduced rates of MVPA amongst glaucoma subjects, FoF and driving status were independently added to model 1 (producing Models 2 and 3 respectively, Table 2). When FoF was added to this model, the RR describing the association between severity of VF loss and MVPA increased from 0.74 to 0.78, and VF loss severity remained associated with less MVPA at a statistically significant level (p < 0.01). When driving status was added to the model, the RR describing the association between severity of VF loss and MVPA decreased from 0.74 to 0.70, and VF loss severity remained associated with less MVPA at a statistically significant level (p < 0.01).

**Table 2:** Multivariate Analysis Exploring the Mediators of Visual Field Loss and Moderate-to-Vigorous Physical Activity in Glaucoma

Variable	Interval	Model 0:	Model 1:	Model 2:	Model 3:
		Univariate Models^a^	Multivariate Models	Model 1+ FoF	Model 1+ Driving
VF loss MD	5 db worse	**0.89 (0.71, 0.89)**	**0.74 (0.67, 0.82)**	**0.78 (0.70, 0.89)**	**0.70 (0.63, 0.79)**
Fear of Falling^b^	Person measure score	**1.3 (1.2, 1.40)**	--	**1.17 (1.08, 1.27)**	**--**
Limited driving	vs Driving without limitation	0.80 (0.54, 1.19)	--	**--**	1.43 (1.00, 2.04)
Not driving	vs Driving without limitation	0.98 (0.64, 1.49)	--	**--**	**2.04 (1.32, 3.15)**
Age	5 y older	**0.60 (0.51, 0.71)**	**0.65 (0.56, 0.76)**	**0.67 (0.58, 0.78)**	**0.63 (0.54, 0.74)**
Female	vs Male	0.74 (0.53, 1.03)	1.01 (0.75, 1.36)	1.27 (0.92, 1.75)	0.86 (0.64, 1.16)
AA	vs Non-AA	**0.28 (0.20, 0.39)**	**0.40 (0.28, 0.57)**	**0.37 (0.26, 0.52)**	**0.35 (0.25, 0.49)**
Education	4 y less	**2.13 (1.71, 2.64)**	1.20 (0.97, 1.48)	1.17 (0.96, 1.44)	1.18 (0.96, 1.45)
Comorbidity	1 more Illness	**0.69 (0.62, 0.76)**	**0.78 (0.71, 0.86)**	**0.89 (0.80, 0.99)**	**0.78 (0.71, 0.86)**

*MVPA* = moderate-to-vigorous physical activity; *VF* = visual field, *MD* = mean deviation; better eye; y = years, *FoF* = fear of falling; *vs* = versus; *AA* = African-American

Values in bold indicate p < 0.05

^a^MVPA is the dependent variable in each model. Model 0 contains bivariate models of each covariate with MVPA. Model 1 contains CS, Age, Sex, Race, Education, and Comorbidity as independent variables. Model 2 adds FoF as a possible mediator between CS and MVPA

^b^Person measure scores are derived from Rasch analytic model. Higher scores indicate greater ability, or less fear of falling

For AMD subjects, decreased CS was associated with less MVPA in univariate and multivariate models (Table 3, Models 0 and 1). When FoF was added to the model, the RR describing the association of CS and MVPA increased from 0.89 to 1.02, and CS loss was no longer associated with MVPA at a statistically significant level (p = 0.53). When driving was added to the model, the RR describing the association between CS and MVPA increased from 0.89 to 0.93, and CS remained associated with less MVPA at a significant level (p = 0.04).

**Table 3:** Multivariate analysis exploring the mediators of contrast sensitivity and moderate-to-vigorous physical activity in age-related macular degeneration

Variable	Interval	Model 0:	Model 1:	Model 2:	Model 3:
		Univariate Models^a^	Multivariate Models	Model 1+ FoF	Model 1+ Driving
Log CS, binocular	0.1 Log units worse	**0.86 (0.80, 0.91)**	**0.89 (0.83, 0.95)**	1.02 (0.95, 1.10)	**0.93 (0.86, 0.99)**
FoF^b^	Person measure score	**1.56 (1.41, 1.73)**	--	**1.70 (1.51, 1.93)**	**--**
Limited driving	vs Driving without limitation	0.71 (0.45, 1.10)	--	**--**	0.92 (0.56, 1.52)
Not driving	vs Driving without limitation	**0.37 (0.22, 0.61)**	--	**--**	**0.40 (0.22, 0.74)**
Age	5 y older	1.07 (0.90, 1.28)	**1.58 (1.28, 1.96)**	1.18 (0.95, 1.46)	**1.69 (1.38, 2.08)**
Female	vs Male	**0.40 (0.27, 0.59)**	**0.33 (0.22, 0.50)**	1.25 (0.81, 1.93)	**0.33 (0.22, 0.49)**
AA	vs Non-AA	**0.12 (0.02, 0.58)**	0.41 (0.07, 2.46)	0.30 (0.05, 1.84)	0.45 (0.08, 2.49)
Education	4 y less	**2.4 (1.6, 3.6)**	**2.18 (1.45, 3.26)**	**3.94 (2.57, 6.02)**	**2.44 (1.61, 3.69)**
Comorbidity	1 more Illness	**0.85 (0.87, 0.96)**	**0.73 (0.64, 0.83)**	**0.81 (0.71, 0.93)**	**0.79 (0.70, 0.90)**

*MVPA* = moderate-to-vigorous physical activity; *CS* = contrast sensitivity; *y* = years; *FoF* = fear of falling; *vs* = versus; *AA* = African-American

Values in bold indicate p < 0.05

^a^MVPA is the dependent variable in each model. Model 0 contains bivariate models of each covariate with MVPA. Model 1 contains CS, Age, Sex, Race, Education, and Comorbidity as independent variables. Model 2 adds FoF as a possible mediator between CS and MVPA

^b^Person measure scores are derived from Rasch analytic model. Higher scores indicate greater ability, or less fear of falling

## Discussion

Among individuals with vision loss from AMD, the association between CS and physical activity in AMD subjects was no longer significant once FoF was added to the model, suggesting that FoF may partially explain the relationship between eye disease and physical activity restriction. However, amongst glaucoma subjects, VF loss remained a statistically significant predictor of physical activity once FoF was added to the model, suggesting that there may be other factors that are important in mediating the relationship between VF loss and physical activity restriction in glaucoma patients. Although we must be careful not to overstate conclusions about mediation from cross-sectional analyses, identifying mediators of decreased physical activity can help in developing targets for intervention. Our data are consistent with a conceptual model that eye disease acquired at an older age results in FoF, which may then cause or exacerbate physical activity restriction, though this relationship may vary with different types of vision loss.

We found that moderate-to-severe FoF compared to mild-to-absent FOF was associated with 4 to 8 times less time spent in MVPA in glaucoma and AMD groups, respectively. This finding supports the existing evidence from other adult populations that FoF is associated with physical activity restriction. For example, several studies relying on self-report of FoF and physical activity found that up to 66 % of community-dwelling older adults restrict engagement in physical activities due to FoF alone [13, 49, 50]. In addition to studies relying upon self-report of FoF and physical activity, those which directly measured physical activity with accelerometers found that decreased MVPA is associated with FoF in diverse populations ranging from those with dual sensory (hearing and vision) impairment [51] and community dwelling older men [52]. Thus, it is clear that FoF is a marker of activity restriction amongst those who are visually impaired individuals. Further work should include measurements of other types of sensory impairments, such as hearing loss, in order to better elucidate whether the mediation effect of FoF is purely due to visual impairment or whether visual impairment is a surrogate for dual- or pan-sensory decline within the same individual [53].

Our results support the prior finding by Wang et. al. that FoF potentially mediates the relationship between vision loss and physical activity restriction [28]. In that study, the authors found that patients with AMD, Fuchs, and glaucoma were more likely to respond that they had restricted their activity as a result of FoF. The authors noted, however, that a limitation of their study was that activity restriction due to FoF was based upon a single self-reported yes/no question, and that activity and FoF were not separately measured. One strength of our study is that we measured FoF using a validated questionnaire, which allowed for objective, linear scaling of responses to questions about fear during tasks of varying difficulty [29]. Additionally, rather than relying upon subjective reports of decreased activity, we measured activity with accelerometers. Self-report is often not the ideal method of capturing true physical activity, as it has been shown to be poorly correlated with accelerometer-defined physical activity (correlation coefficients of 0.2-0.4) [54–57]. Previous work has also demonstrated that objectively-measured physical activity is more closely associated with physical characteristics such as elevated BMI, triglycerides and blood sugar than self-report of physical activity [58]. Hence, self-reported and accelerometer-measured physical activity may be measuring different constructs, with the former measuring one’s perception of functionality while the latter captures actual physical activity level. Therefore, we believe that the current study better characterizes the potential intermediary role of FoF between vision loss and real-world physical activity.

A surprising finding from the current study is that the relationship between VF loss and physical activity amongst glaucoma patients remained significant after adding FoF to the model, and that the magnitude of this association decreased by only 15 %. One possibility is that many glaucoma patients begin restricting their physical activity as a result of vision-related balance or gait deficits even before FoF sets in. Alternately, it is possible that our cross-sectional model was not adequate for capturing the true impact of FoF on physical activity restriction. Finally, other unknown factors may serve as mediators between VF and physical activity.

Driving was also investigated for its ability to attenuate the relationship between vision loss and physical activity, given that driving is a primary method for leaving the home and that rates of physical activity are significantly higher outside the home than within the home [30]. The fact that driving status did not act as potential mediator in our current work may be explained by the fact that time spent away from home is not strongly predicted by driving status [59]. For example, persons who lose their driving ability may still find adaptive methods of leaving the home through relying on others for transportation. Additionally, those who are unable to leave their home may adapt by performing more physical activity inside the home, which could diminish the attenuation effect of driving.

The identification of FoF as a possible intermediary in reducing physical activity among AMD patients establishes FoF as a possible target for preventing physical activity decline in this group. A substantial number of interventional studies in the past decade which have been targeted at reducing FoF with Tai Chi or other forms of exercise have mostly proven successful [60–62]. Since prior works have identified depression and anxiety [63, 64], poor balance [43], visual impairment [65], and an unsafe environment [66] as risk factors for FoF, rehabilitation strategies may be effective by targeting these factors as well. Further study should focus upon establishing which of these elements are most critical to reducing FoF, and testing multifaceted approaches which address FoF in this patient population.

A limiting feature of this study was the cross-sectional design, precluding definitive conclusions about mediation given that a longitudinal design is necessary to establish temporality of FoF, vision loss, and physical activity restriction. Secondly, although our study size was powered to detect differences in physical activity levels between groups, it is difficult to ascertain whether our sample size is appropriate to detect potential mediation of certain variables on physical activity. Generalizability of our results may also be limited as a result of our selection of individuals who receive their care from an urban tertiary care facility. Our urban population may differ from a rural population wherein lifestyles differences predispose individuals to different levels of physical activity. We also faced the potential for selection bias since patients with the greatest physical activity restriction may have been less likely to participate in the study given the need for additional study visits, although participation was encouraged by allowing patients to complete their testing on the same day as their clinical visits. Additionally, although comorbid illnesses were measured in this study and included as covariates in our model, the size of our dataset is not large enough to allow for us to weight individual illnesses for their impact upon physical activity. Finally, while the technology used to evaluate mobility provided reliable and quantifiable measurements, a limitation of accelerometers is that they do not accurately calculate the calories burned during activities such as cycling and swimming. Despite this limitation, there is a large body of evidence demonstrating that accelerometers do capture true physical activity levels significantly better than questionnaires [58].

## Conclusion

Our findings lend support to the hypothesis that FoF is a marker for physical activity restriction amongst both glaucoma and AMD patients, and that it may partially mediate the association between vision loss and physical activity restriction amongst AMD patients. The importance of FoF is well documented, and our findings indicate that it may be a key factor along the pathway from vision loss to decreased physical activity. Our work emphasizes the importance of interventions aimed at reducing FoF to reduce the sedentary lifestyle associated with vision loss. Future work should assess the safety and effectiveness of strategies which will reduce severity of FoF while also making an effort to replace inactivity with low-risk physical activity.

## Additional file

Additional file 1:
**University of Chicago Fear of Falling Measure.**

## Abbreviations

FoF: Fear of falling
VF: Visual field
CS: Contrast sensitivity
AMD: Age-related macular degeneration
VA: Visual acuity
dB: decibels
MD: Mean deviation
SITA: Swedish interactive thresholding algorithm
GHT: Glaucoma hemifield test
SEEDS: Salisbury Eye Evaluation Driving Study
EDTRS: Early Treatment of Diabetic Retinopathy Study
logMAR: logarithm of the minimum angle of resolution
logCS: Contrast sensitivity converted to a log scale
PCO: Posterior capsular opacification
BMI: Body mass index
MMSE: Mini Mental State Exam
MVPA: Moderate-to-vigorous physical activity
RR: Rate ratio
IQR: Interquartile range
