# Supplementary material for: Physical activity restriction in age-related eye disease: a cross-sectional study exploring fear of falling as a potential mediator
Source: BMC Geriatr. 2015 Jun 12;15:64. doi: 10.1186/s12877-015-0062-8 (PMC4464712; doi:10.1186/s12877-015-0062-8)
Supplement: Additional file 1: — University of Chicago Fear of Falling Measure. [file 12877_2015_62_MOESM1_ESM.pdf]

University of Illinois at Chicago Fear of Falling Measure

FOF1. If you were to walk outside when icy, how worried are you of falling?
- (1) Very Worried
- (2) Moderately worried/ A little worried
- (3) Not at all worried

FOF2. If you were to carry bundles up poorly lit stairs, how worried are you of falling?
- (1) Very Worried
- (2) Moderately worried/ A little worried
- (3) Not at all worried

FOF3. If you were to use a step stool to reach in kitchen cabinets, how worried are you of falling?
- (1) Very worried
- (2) Moderately worried/ A little worried
- (3) Not at all worried

FOF4. If you were to climb a poorly lit stairs, how worried are you of falling?
- (1) Very worried
- (2) Moderately worried/ A little worried
- (3) Not at all worried

FOF5. If you were to stand on a moving bus, how worried are you of falling?
- (1) Very worried
- (2) Moderately worried/ A little worried
- (3) Not at all worried

FOF6. If you were to climb up bus stairs, how worried are you of falling?
- (1) Very worried
- (2) Moderately worried/ A little worried
- (3) Not at all worried

FOF7. If you were to get in/out of the bathtub, how worried are you of falling?
- (1) Very worried
- (2) Moderately worried/ A little worried
- (3) Not at all worried

FOF8. If you were to carry bundles up a well-lit stairs, how worried are you of falling?
- (1) Very worried
- (2) Moderately worried/ A little worried
- (3) Not at all worried

FOF9. If you were to step off a curb onto the street, how worried are you of falling?
- (1) Very worried
- (2) Moderately worried/ A little worried
- (3) Not at all worried

FOF10. If you were to walk on a crowded sidewalk, how worried are you of falling?
- (1) Very worried
- (2) Moderately worried/ A little worried
- (3) Not at all worried

FOF11. If you were to climb up a well-lit stairs, how worried are you of falling?
- (1) Very worried
- (2) Moderately worried/ A little worried
- (3) Not at all worried

FOF12. If you were to use an escalator, how worried are you of falling?
- 1) Very worried
- (2) Moderately worried/ A little worried
- (3) Not at all worried

FOF13. If you were to take a walk, how worried are you of falling?
- (1) Very worried
- (2) Moderately worried/ A little worried
- (3) Not at all worried

FOF14. If you were to get in/out of a car, how worried are you of falling?
- (1) Very worried
- (2) Moderately worried/ A little worried
- (3) Not at all worried

FOF15. If you were to carry a full plate to the dinner table, how worried are you of falling?
- (1) Very worried
- (2) Moderately worried/ A little worried
- (3) Not at all worried

FOF16. If you were to pick up something lightweight off the floor, how worried are you of falling?
- (1) Very worried
- (2) Moderately worried/ A little worried
- (3) Not at all worried

Adapted from: Velozo C a, Peterson EW: **Developing meaningful Fear of Falling Measures for community dwelling elderly.** Am J Phys Med Rehabil 2001, 80:662–73.
